# Selective Anticancer Therapy Using Pro-Oxidant Drug-Loaded Chitosan–Fucoidan Nanoparticles

**DOI:** 10.3390/ijms20133220

**Published:** 2019-06-30

**Authors:** Dae Gun Choi, Jayachandran Venkatesan, Min Suk Shim

**Affiliations:** 1 Division of Bioengineering, Incheon National University, Incheon 22012, Korea; 2 Yenepoya Research Centre, Yenepoya (Deemed to Be University), Deralakatte, Mangalore 575018, Karnataka, India

**Keywords:** chitosan, fucoidan, nanoparticles, piperlongumine, pro-oxidant cancer therapy

## Abstract

Pro-oxidant therapy exploiting pro-oxidant drugs that can trigger cytotoxic oxidative stress in cancer cells has emerged as an innovative strategy for cancer-specific therapy. Piperlongumine (PL) has gained great interest as a novel pro-oxidant agent, because it has an ability to trigger cancer-specific apoptosis through the increase of oxidative stress in cancer cells. However, the use of PL is limited in the clinic because of its hydrophobic nature. In this study, chitosan- and fucoidan-based nanoparticles were prepared for the effective intracellular delivery of PL into cancer cells. Chitosan and fucoidan formed nanoparticles by ionic gelation. The chitosan- and fucoidan-based nanoparticles (CS–F NPs) effectively encapsulated PL, and increased its water solubility and bioavailability. CS–F NPs showed very low cytotoxicity in human prostate cancer cells, demonstrating its high potential for in vivo applications. The PL-loaded chitosan–fucoidan nanoparticles (PL-CS–F NPs) efficiently killed human prostate cancer cells via PL-induced intracellular reactive oxygen species (ROS) generation. This study demonstrates that CS–F NPs are promising natural polymer-based drug carriers for safe and effective PL delivery.

## 1. Introduction

Chemotherapy is one of the most common types of treatment for cancer, which can be used either alone or in combination with other therapies, such as surgery and radiation therapy [1]. However, many conventional anticancer drugs suffer some limitations, such as poor aqueous solubility, short blood circulation, and lack of cell-specificity [2,3]. Nanoparticle-based drug carriers have attracted great attention in cancer chemotherapy, because they can offer many benefits including an enhanced aqueous solubility of chemodrugs, prolonged blood circulation time, increased cellular uptake, and enhanced accumulation in tumors [4,5].

Various materials, including synthetic polymers, lipids, proteins, polysaccharides, and inorganic nanomaterials, have been employed to prepare nanoparticles [6]. Among them, natural polymers have been widely utilized for the preparation of nanoparticle-based drug carriers, because of their excellent biocompatibility [7]. Chitosan, a natural cationic polymer derived from chitin, has been intensively utilized as a safe drug carrier because of its biocompatible and biodegradable properties [8,9,10]. Previous studies have demonstrated that chitosan-based nanoparticles hold high potential for the safe and efficient delivery of anticancer drugs [8,11].

Recently, pro-oxidant cancer therapy that can selectively kill cancer cells by the generation of cytotoxic oxidative stress in cancer cells has emerged as an effective strategy for cancer-specific treatments [12,13]. Piperlongumine (PL) is a new class of pro-oxidant drug and has been known to induce cancer-specific apoptosis through the elevation of intracellular reactive oxygen species (ROS) [14,15,16]. It has been reported that PL dramatically increases intracellular ROS, and thus induces oxidative stress by interfering with ROS homeostatic regulators such as glutathione S-transferase pi 1 (GSTP1) [17]. Because cancer cells are more sensitive than normal cells to the oxidative stress, the increased ROS by PL can selectively kill cancer cells [18]. Despite the therapeutic potential of PL, its clinical use has been hampered because of its hydrophobicity [19,20]. Therefore, the development of drug carriers that can efficiently encapsulate hydrophobic PL is crucial in order to enhance its anticancer efficacy [19,20].

Here, we prepared natural polymer-based nanoparticles for the safe and efficient intracellular delivery of PL. The PL-loaded nanoparticles were prepared by the ionic crosslinking of chitosan and fucoidan. Fucoidan, an anionic sulfated polysaccharide found mainly in brown seaweed, has received increasing interest in biomedical applications, because of its various bioactivities, including antiviral, anti-inflammatory, and antitumor activities [21]. The electrostatic interactions between anionic fucoidan and cationic chitosan can form the PL-loaded polyelectrolyte nanocomplexes. It is expected that the ionically crosslinked chitosan-fucoidan nanoparticles would stably encapsulate PL with enhanced water solubility. More importantly, the nanoparticles can be used as safe drug carriers, because both chitosan and fucoidan are nontoxic natural polymers. 

**Figure 1:**
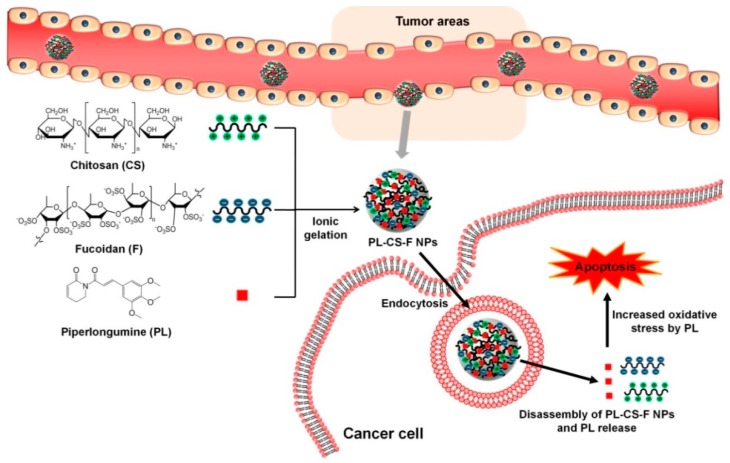
Schematic illustration showing the fabrication of piperlongumine-loaded chitosan–fucoidan nanoparticles (PL-CS–F NPs) and their working mechanisms for apoptosis in cancer cells.

As shown in Figure 1, the ionic crosslinking of chitosan and fucoidan in the presence of PL forms PL-loaded chitosan–fucoidan nanoparticles (PL-CS–F NPs). The nanoparticles can be accumulated at tumor sites by the enhanced permeability and retention (EPR) effect. After internalization in cancer cells via endocytosis, the nanoparticles are disassembled and thus release PL. Then, the released PL can increase intracellular ROS in the cancer cells, thus leading to increased oxidative stress in cancer cells. Because cancer cells are more sensitive to oxidative stress than normal cells [13], the increased oxidative stress can trigger cancer-specific apoptosis. The physicochemical properties and drug encapsulating efficiency of the nanoparticles were characterized. In addition, the cancer-specific cytotoxicity of PL-loaded nanoparticles was investigated in vitro.

## 2. Results and Discussion

### 2.1. Characterization of PL-CS–F NPs

**Figure 2:**
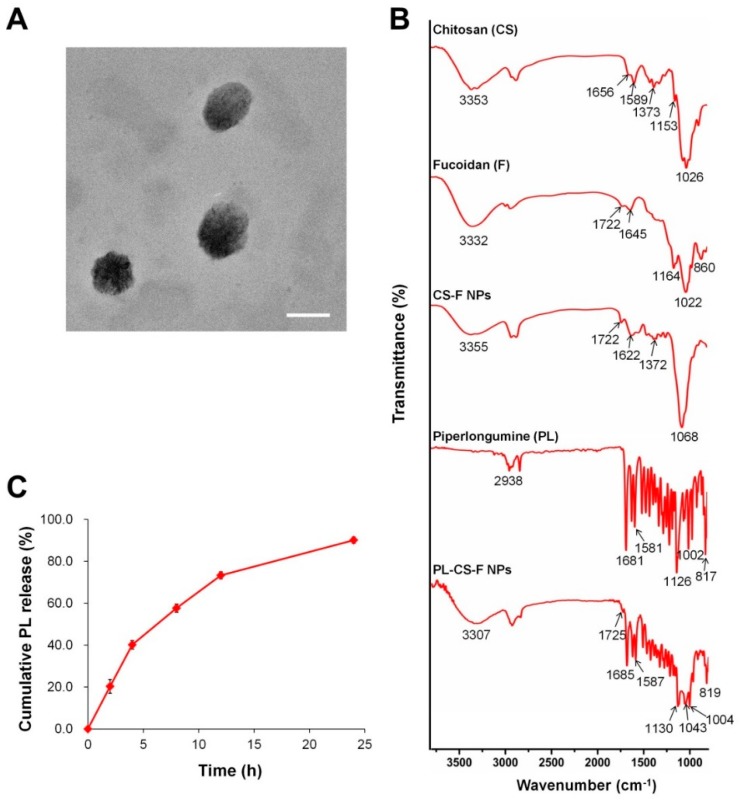
(**A**) TEM image of PL-CS–F NPs. Scale bar = 200 nm. (**B**) FT-IR spectra of chitosan, fucoidan, CS–F NPs, PL, and PL-CS–F NPs. (**C**) Drug release profiles of PL-CS–F NPs in phosphate buffered saline (PBS) (pH 7.4) at 37 °C for 24 h.

CS–F NPs and PL-CS–F NPs were prepared by ionic gelation in the acidic condition (around pH 5.0), because protonated chitosan in the acidic condition can be efficiently complexed with negatively charged fucoidan. The prepared CS–F NPs and PL-CS–F NPs were separated from the unloaded PL by centrifugation. The pellet of the NPs was dissolved in ethanol, and the amount of PL in the solution was determined by an absorbance measurement at 329 nm. PL-CS–F NPs successfully encapsulated PL, resulting in 16.87% PL encapsulation efficiency (Table 1). After the purification process, nano-sized and positively charged CS–F NPs and PL-CS–F NPs were obtained. The sizes and zeta potentials of the CS–F NPs and PL-CS–F NPs are represented in Table 1. The morphology of the self-assembled PL-CS–F NPs was observed by TEM. The TEM images demonstrate that the shape of PL-CS–F NPs was spherical, with an average diameter of 200 nm (Figure 2A). 

The formation of PL-loaded CS–F NPs by ionic gelation was also examined by Fourier transform infrared (FT-IR) spectroscopy. The FT-IR spectra of the chitosan, fucoidan, CS–F NPs, PL, and PL-CS–NPs are represented in Figure 2B. 

The FT-IR spectra of the chitosan showed a strong and broad peak at 3353 cm^−1^, which corresponds to the O–H vibration. Chitosan also showed characteristic peaks of NH_2_ bending and C=O stretching of the amide at 1589 cm^−1^ and 1656 cm^−1^, respectively. The peaks at 1373 cm^−1^, 1153 cm^−1^, and 1026 cm^−1^ are associated with the C–N stretching, asymmetric C–O–C stretching, and C–O skeletal vibration of the chitosan, respectively [22]. Fucoidan exhibited the characteristic peaks at 1164 cm^−1^ and 860 cm^−1^, which correspond to the S=O asymmetric stretching and C–O–S stretching of the sulfate groups, respectively (Figure 2B) [23]. The spectrum of the CS–F NPs showed the characteristic peaks from the chitosan (e.g., 1371 cm^−1^) and fucoidan (e.g., 1722 cm^−1^), thus indicating that the CS–F NPs were formed by the electrostatic interactions of chitosan and fucoidan. In the spectra of the PL-CS–F NPs, the characteristic peaks of the PL and CS–F NPs were observed, thus demonstrating that PL was successfully encapsulated in the CS–F NPs. 

### 2.2. Drug Release of PL-CS–F NPs

The PL release rate from the PL-CS–F NPs in phosphate buffered saline (PBS) (pH 7.4) was assessed. As shown in Figure 2C, the PL-CS–F NPs exhibited a sustained release of PL in PBS, without showing any burst effects. Approximately 90% of the encapsulated PL was released from the NPs after 24 h of incubation at pH 7.4 (Figure 2C). More sustained drug release would be achieved by using higher molecular weights of chitosan and fucoidan for the formation of PL-CS–F NPs.

### 2.3. Cancer-Specific Cytotoxicity of PL-CS–F NPs

**Figure 3:**
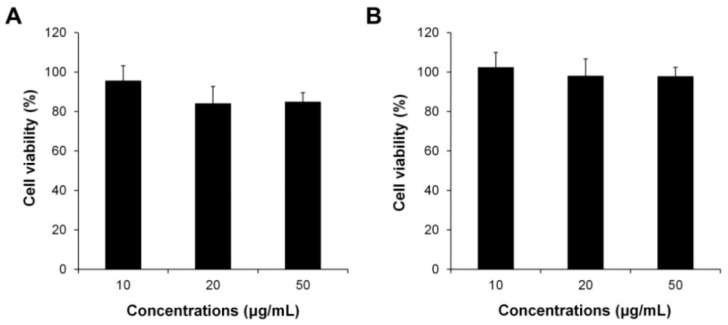
Cytotoxicity evaluation of CS–F NPs against (**A**) hDFB and (**B**) PC-3 cells at different concentrations.

To demonstrate the low toxicity of natural polymer-based CS–F NPs, the cell viabilities of the CS–F NPs against human dermal fibroblast cells (hDFB) and human prostate cancer cells (PC-3) were evaluated using a 3-(4,5-dimethylthiazol-2-yl)-2,5-diphenyl tetrazolium bromide (MTT) assay. The cell viability of plain CS–F NPs without PL was higher than 85% in both the hDFB and PC-3 cells, even at the highest concentration tested (i.e., 50 μg/mL; Figure 3). This result indicates that natural polymer-based CS–F NPs are low-toxic and suitable as a safe drug carrier.

**Figure 4:**
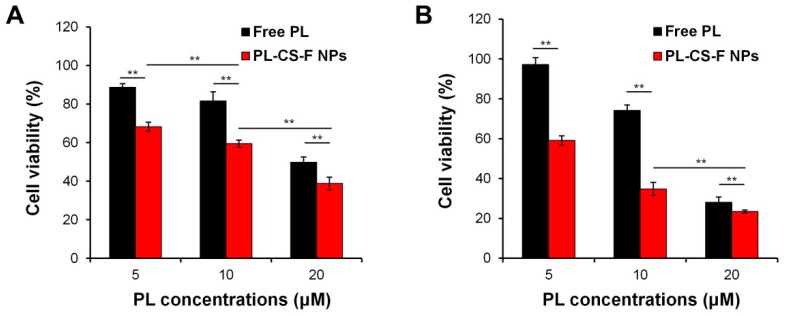
Cytotoxicity evaluation of free PL and PL-CS–F NPs against (**A**) hDFB and (**B**) PC-3 cells at different concentrations of PL (** *p* < 0.01).

The selective anticancer activity of the PL–CS–F NPs was clearly confirmed (Figure 4). Both the free PL and PL-CS–F NPs showed a higher cytotoxicity against PC-3 cells than hDFB cells in a dose-dependent manner (Figure 4). In addition, the PL-CS–F NPs showed a higher cytotoxicity than the free PL did at equivalent PL concentrations, regardless of the cell types. This might be attributed to the efficient PL release by the PL-CS–F NPs. These results indicate that PL-CS–F NPs are effective and safe nanocarriers for cancer-specific pro-oxidant therapy.

### 2.4. Elevation of Intracellular ROS by PL-CS–F NPs 

**Figure 5:**
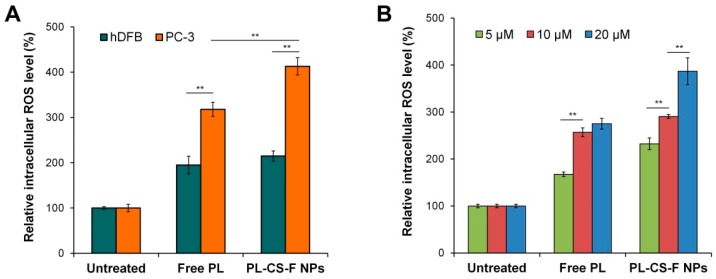
(**A**) Quantification of relative intracellular reactive oxygen species (ROS) levels in hDFB and PC-3 cells after treatment with free PL (25 μM) and PL-CS–F NPs (25 μM PL). The intracellular ROS levels of the untreated cells were normalized to 100% (** *p* < 0.01). (**B**) Relative intracellular ROS levels in PC-3 cells after treatment with different concentrations of PL. The intracellular ROS levels of the untreated cells were normalized to 100% (** *p* < 0.01).

The intracellular ROS levels of the PC-3 and hDFB cells treated with free PL, CS–F NPs, or PL-CS–F NPs were quantified by using 2′,7′-dichlorofluorescein diacetate (DCF-DA), a cell permeable ROS-responsive dye. As shown in Figure 5A, the free PL significantly increased the intracellular ROS levels compared with the untreated cells, as previously reported [11,12]. Likewise, the PC-3 cells treated with PL-CS–F NPs revealed a dramatic increase in ROS levels. These results demonstrate that PL induces the increase of intracellular ROS in cancer cells, and that CS–F NPs can facilitate the intracellular delivery of PL. It has been reported that PL selectively induces apoptotic death in cancer cells by increasing oxidative stress in the cells [14,15]. Therefore, it is speculated that the high cytotoxicity of free PL and PL-CS–F NPs in PC-3 cells (Figure 4B) is closely related to the elevated intracellular ROS levels induced by PL.

It is noteworthy that the PC-3 cells treated with free PL or PL-CS–F NPs showed significantly higher intracellular ROS levels compared with the hDFB cells treated with free PL or PL-CS–F NPs (Figure 5A). This result clearly indicates that PL selectively induces oxidative stress in cancer cells over normal cells, thus leading to cancer-specific apoptosis. The lower intracellular ROS levels in the hDFB cells might be ascribed to their ability for counterbalancing ROS production via antioxidant systems [24]. In contrast to normal cells, cancer cells such as PC-3 cells are not able to preserve the redox balance after treatment with pro-oxidant PL. Similar results have been reported from other studies [15,25]. The dose-dependent increase of intracellular ROS levels was also confirmed. As shown in Figure 5B, the PC-3 cells treated with free PL and PL-CS–F NPs exhibited higher levels of intracellular ROS when treated with higher concentrations of PL. This result clearly indicates that the dose-dependent cytotoxicity of PL-CS–F NPs (Figure 4B) is mainly attributed to their dose-dependent intracellular ROS production. 

### 2.5. Apoptosis of PL-CS–F NPs

**Figure 6:**
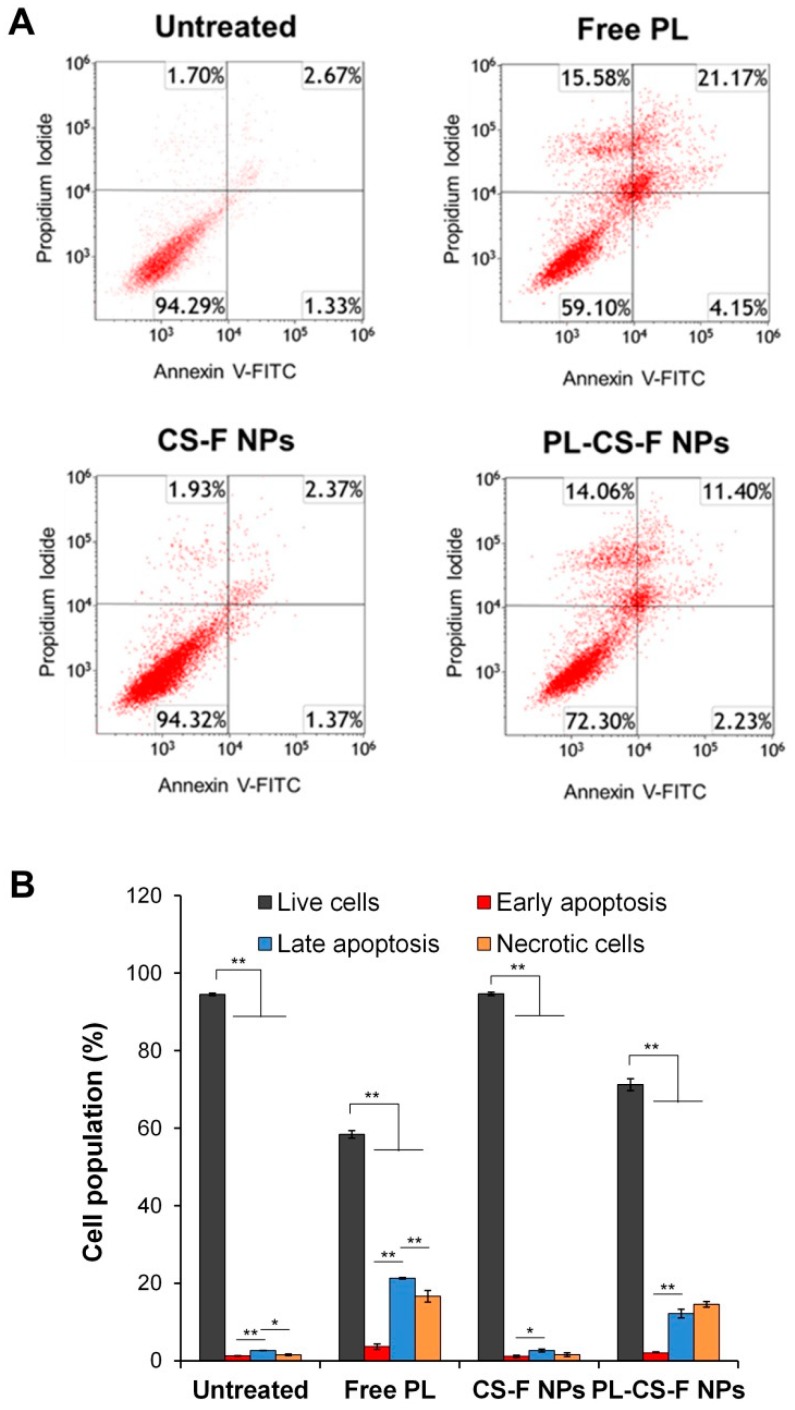
Apoptosis of PC-3 cells treated with PL-CS–F NPs. (**A**) Flow cytometric analysis of PC-3 cells treated with free PL (10 μM), CS–F NPs, and PL-CS–F NPs (10 μM PL). (**B**) Quantification of live, early apoptotic, late apoptotic, and necrotic cells from the flow cytometric histograms (* *p* < 0.05, ** *p* < 0.01).

To investigate whether PL-induced ROS generation contributes to apoptosis in cancer cells, the apoptosis of PC-3 cells was examined after treatment with free PL, plain CS–F NPs, and PL-CS–F NPs, respectively. The apoptosis was confirmed by flow cytometry with Annexin V-fluorescein isothiocyanate (FITC)/propidium iodide (PI) staining. As shown in Figure 6, the plain CS–F NPs showed a very low level of late apoptosis in the PC-3 cells (2.37% in the upper right quadrant). Importantly, both the free PL and PL-CS–F NPs significantly increased the percentage of late apoptotic cells compared with the untreated cells (Figure 6A,B). Taken together with the results from the intracellular ROS generation analysis (Figure 5), this apoptosis result implies that the elevated level of intracellular ROS induced by PL-CS–F NPs is a crucial factor for the dramatic increase of apoptosis in PC-3 cells treated with PL-CS–F NPs.

## 3. Materials and Methods

### 3.1. Materials

Chitosan, fucoidan, and 2′,7′-dichlorofluorescein diacetate (DCF-DA) were purchased from Sigma-Aldrich (St. Louis, MO, USA). Piperlongumine was supplied from Cayman Chemical (Ann Arbor, MI, USA). hDFB human dermal fibroblast cells and PC-3 human prostate cancer cells were obtained from ATCC (Manassas, VA, USA). The AnnexinV-FITC/PI staining kit was purchased from BD Life Sciences (San Jose, CA, USA). Tween 80 was purchased from Duksan Pure Chemicals (Ansan-si, Gyunggido, Korea).

### 3.2. Preparation of CS–F NPs and PL-CS–F NPs

To yield 2.5 mg/mL of the chitosan solution, 250 mg of chitosan was dissolved in 100 mL of 1% (*v*/*v*) acetic acid solution along with 0.25% (*v*/*w*) Tween 80 (0.25 mL). The chitosan solution was stirred continuously until most of the chitosan was fully dissolved. The undissolved chitosan was filtered through a 0.45 μm polyvinylidene fluoride (PVDF) syringe filter. Then, 50 mg of fucoidan was dissolved in 100 mL of deionized (DI) water, 2 mL of the chitosan solution (2.5 mg/mL) was stirred at 500 rpm, and 4 mL of the fucoidan solution (0.5 mg/mL) was added to 2 mL of the chitosan solution to form chitosan–fucoidan polyelectrolyte complex nanoparticles (CS–F NPs). The mixture was stirred for 1.5 h, and the supernatant was removed by centrifugation at 12,000 rpm for 5 min.

To prepare the PL-loaded chitosan–fucoidan nanoparticles (PL-CS–F NPs), 2 mL of chitosan solution (2.5 mg/mL) containing 0.25% (*v*/*w*) Tween 80 was mixed with 1 mL of PL solution in ethanol (2 mg/mL). Then, 4 mL of aqueous fucoidan solution (0.5 mg/mL) was slowly added to form the PL-chitosan–fucoidan polyelectrolyte complex nanoparticles (PL-CS–F NPs). The mixture was stirred for 1.5 h, and then the supernatant was removed using centrifuge 12,000 rpm for 5 min.

### 3.3. Characterization of PL-CS–F NPs

The size and zeta potential of the PL-CS–F NPs were monitored by dynamic light scattering analysis (DLS; Nano-ZS, Malvern, Worcestershire, U.K.). The FT-IR spectra of the CS–F NPs and PL-CS–F NPs were recorded using a Bruker Vertex 80v spectrometer (Bruker Optics, Ettlingen, Germany). The morphology of the PL-CS–F NPs was observed by transmission electron microscopy (TEM). Droplets of an aqueous suspension of PL-CS–F NPs were deposited onto a carbon-coated copper grid (Ted Pella, Redding, USA) and dried under ambient conditions. The TEM images were examined with a TALOS F200X microscope (FEI, Hillsboro, OR, USA).

### 3.4. Quantification of PL Encapsulation Efficiency

To quantify the PL encapsulation efficiency of the PL-CS–F NPs, they were centrifuged at 12,000 rpm to separate the pellet of the NPs from the supernatant (containing unloaded PL). The pellet of the NPs was rinsed with DI water three times. The amount of PL in the NPs was quantified using UV-VIS spectroscopy at 329 nm. Briefly, the pellet of the PL-CS–F NPs was dispersed in DI water, and 100 volumes of ethanol were added to dissolve the NPs. The encapsulation efficiency was determined using the following formula. 

Encapsulation efficiency (%) = (Amount of drug in NPs/Initial amount of drugs) × 100(1)

### 3.5. Drug Release Study of PL-CS–F NPs

The PL-CS–F NPs were incubated in PBS (pH 7.4) at 37 °C for various periods of time. At a predetermined interval, the sample solution (0.5 mL) was centrifuged at 12,000 rpm. Then, the supernatant containing the released PL was mixed with 100 volumes of ethanol to dissolve the remained PL in the supernatant. The released PL in the supernatant was quantified using UV-VIS spectroscopy at 329 nm. 

### 3.6. Cell Culture

hDFB human dermal fibroblast cells and PC-3 human prostate cancer cells were used to determine the anticancer activities of the PL-CS–F NPs. The hDFB cells were cultured in Dulbecco’s Modified Eagle Medium (DMEM) containing 10% fetal bovine serum (FBS) and 1% penicillin/streptomycin. The PC-3 cells were cultivated in Rosewell Park Memorial Institute Medium (RPMI 1640) containing 10% FBS and 1% penicillin/streptomycin. They were maintained at 37 °C in a 5.0% CO_2_ humidified atmosphere.

### 3.7. Assessment of In Vitro Cytotoxicity

To evaluate the anticancer activities of the PL-CS–F NPs, a conventional MTT assay was conducted. Briefly, hDFB and PC-3 cells were seeded into 96-well plates at a density of 1 × 10^4^ cells per well and incubated for 24 h. Then, the cells were treated with a serum-free medium containing plain CS–F NPs, PL-CS–F NPs, or free PL. The concentrations of PL in the PL-CS–F NPs were set at 5, 10, and 20 μM. The untreated cells were used as a blank control. After the cells were incubated with the samples for 4 h, the medium was replaced with 10% FBS-containing culture medium. After the cells were incubated for 24 h, the medium was replaced with 100 μL of the MTT solution in a serum-free medium (1 mg/mL). The cells were incubated for 4 h at 37 °C to generate formazan crystals from live cells. The MTT solutions were removed, and the crystals were dissolved in DMSO. The absorbance was measured at 565 nm with a microplate reader (Infinite M200 pro, TECAN, Grödig, Austria). 

### 3.8. Quantification of Intracellular ROS Levels

The PC-3 cells and hDFB cells were cultured into a 12-well plate at a density of 2 × 10^5^ cells per well for the quantification of intracellular ROS levels by using ROS-sensitive DCF-DA). After the cells were incubated for 24 h, they were treated with 10 μM DCF-DA solution in PBS. After 30 min of incubation, the cells were washed with PBS and treated free PL (25 μM) and PL-CS–F NPs (25 μM PL), respectively. After 4 h of incubation, the cells were washed with PBS two times. The fluorescence intensity of the cells was quantified using a flow cytometry with FITC channel (CytoFLEX, Beckman Coulter, Brea, CA, USA).

### 3.9. Apoptosis Analysis by Annexin V-FITC/PI Staining

The PC-3 cells were cultured into a 12-well plate at a density of 1 × 10^5^ cells per well for the apoptosis analysis. After the cells were incubated for 24 h, they were treated with free PL (10 μM), plain CS–F NPs (10 μM PL), and PL-CS–F NPs (10 μM PL), respectively. After 4 h of incubation, Annexin V-FITC and propidium iodide (PI) staining was performed, according to the manufacturer’s protocol (BD Biosciences, Heidelberg, Germany).

### 3.10. Statistical Analysis

The data were expressed as mean ± standard deviation values. One-way ANOVA was carried out to determine statistical significance between different groups.

## 4. Conclusions

PL-CS–F NPs were synthesized by the ionic gelation cross-linking of chitosan and fucoidan. The PL-CS–F NPs could efficiently encapsulate hydrophobic PL. The CS–F NPs showed a very low cytotoxicity against both hDFB and PC-3 cells, indicating that CS–F NPs are low-toxic. The PL-CS–F NPs showed a higher cytotoxicity in the PC-3 cells than in the non-cancerous hDFB cells, demonstrating their cancer-specific anticancer effects. In addition, the PL-CS–F NPs significantly increased the intracellular ROS levels in the PC-3 cells, thus inducing apoptosis in the cells. These findings suggest that PL-CS–F NPs are safe and effective for the intracellular delivery of PL for cancer-specific pro-oxidant therapy. Further chemical modification of PL-CS–F NPs to avoid the nonspecific binding of plasma proteins is currently under investigation, which is indispensable to enhance their in vivo pharmacokinetic properties.

## Figures and Tables

**Table 1:** Size, zeta potentials, polydispersity index (PDI), and piperlongumine (PL) encapsulation efficiency of chitosan–fucoidan nanoparticles (CS–F NPs) and PL-loaded CS–F NPs (PL-CS–F NPs).

Samples	Size (nm)	PDI	Zeta Potential (mV)	Encapsulation Efficiency of PL (%)
CS–F NPs	234.73 ± 12.82	0.162 ± 0.004	7.86 ± 0.72	N/A
PL-CS–F NPs	215.70 ± 13.38	0.163 ± 0.030	19.26 ± 2.02	16.87 + 1.43
